# Exclusive enteral nutrition for induction of remission in pediatric Crohn's disease: Short‐ and long‐term tolerance and acceptance

**DOI:** 10.1002/jpr3.12163

**Published:** 2024-12-30

**Authors:** Catto Sandrine, Dugelay Emmanuelle, Viala Jérôme, Christine Martinez‐Vinson

**Affiliations:** ^1^ Department of Dietetics, Hôpital Universitaire Robert Debré, Assistance Publique Hôpitaux de Paris Université de Paris Paris France; ^2^ Department of Pediatric Gastroenterology, Hôpital Universitaire Robert Debré, Assistance Publique Hôpitaux de Paris Université de Paris Paris France

**Keywords:** children, inflammatory bowel disease, nutrition

## Abstract

**Objectives:**

In children with mild to moderate Crohn's disease (CD), exclusive enteral nutrition (EEN) is the first‐line treatment. However, adherence to this therapeutic strategy remains challenging because of numerous psychosocial factors. This study aimed to evaluate the short‐term acceptability and long‐term tolerance of EEN.

**Methods:**

A single‐center retrospective study involving a pediatric population with CD was conducted at Robert‐Debré Hospital in Paris between December 2023 and March 2024.

**Results:**

Thirty‐two patients responded to the questionnaire. developed specifically for this study. It included detailed sections on the EEN received, duration, observed consequences, difficulties encountered by patients and their families, and sociodemographic information. Twenty patients (62%) received oral treatment, while 12 (38%) required a nasogastric tube (NGT). Thirty‐eight percent of the patients prematurely discontinued treatment. Most children reported difficulties related to taste, vomiting, and discomfort caused by the NGT. Fifty‐nine percent of children would recommend treatment due to its rapid effectiveness, despite the challenges posed by the taste and exclusive nature of the diet. Thirty‐two percent of patients reported persistent eating disorders (EDs) long after treatment discontinuation, and 12.5% reported social disorders. Despite strict treatment constraints, children managed to adapt and maintain their daily activities.

**Conclusion:**

EEN has significant benefits for children with CD; however, its acceptability is mixed owing to dietary and social constraints. Adequate dietary and psychological support is crucial for improving adherence to treatment and preventing EDs in one third of our patients after treatment.

## Abstract (highlights)

**What is Known:**

- Exclusive enteral nutrition (EEN) is recommended as the first‐line treatment to induce remission in pediatric Crohn's disease.
- Adherence to EEN is challenging.

**What is New:**

- Thirty‐two percent of patients reported persistent eating disorders (EDs) long after stopping EEN, and 12.5% had social disorders.
- Psychological support during EEN could avoid long‐term EDs.

## 1 INTRODUCTION

Since the 1970s, numerous studies have demonstrated the benefits of exclusive enteral nutrition (EEN) for the management of pediatric Crohn's disease (CD). Since 2014, the European Crohn's and Colitis Organization and the European Society of Pediatric Gastroenterology, Hepatology, and Nutrition have recommended the use of EEN as a first‐line treatment for luminal CD in children.^1^, ^2^ They recommend a 6‐ to 8‐week course of EEN to achieve clinical and biological remission, providing 120% of the theoretical resting energy expenditure as induction therapy. However, adherence to EEN remains challenging owing to various psychosocial factors.^3^ The duration of treatment and requirement for exclusive liquid nutrition affect the patient's quality of life,^4^ leading to compliance difficulties documented in numerous studies.^4^, ^5^, ^6^, ^7^, ^8^ Food monotony, discomfort from the use of a nasogastric tube (NGT), and aversion to the taste of oral EEN can occur.^9^, ^10^, ^11^ It is important to note that specific feeding difficulties associated with this treatment are frequently reported, but no studies have been conducted on this subject. This study aimed to evaluate the short‐term acceptability and long‐term tolerance of EEN, particularly in the development of eating disorders (EDs).

## 2 METHODS

This retrospective, single‐center study, conducted at Robert‐Debré Hospital in Paris between December 2023 and March 2024 (4 months), involved a pediatric population with CD. The study was based on a questionnaire developed specifically for this study. This questionnaire was developed by the pediatric gastroenterology team, including the referring nutrition physician, the referring inflammatory bowel disease (IBD) physician, and the team of dietitians–nutritionists responsible for these conditions. It included detailed sections on the EEN received, duration, observed consequences, difficulties encountered by patients and their families, and sociodemographic information. All CD patients in active follow‐up who had received EEN were given the questionnaire by email for those followed in outpatient clinics and face‐to‐face for those followed in day hospital. Clinical parameters were retrospectively collected from clinical charts.

Approval was obtained from the local ethics committee (reference number: 2022‐314bis). All patients and their guardians have consented to our informed consent statement. Consent was verbal.

### 2.1 Questionnaire

The questionnaire contained questions relating to:

General information: Age, sex, presence of siblings at home.

EEN details: Age at onset, initial use, duration, place of initiation, mode of administration, dietary consultation, continuity of family meals, modalities of administration, premature discontinuation, and reasons.

Difficulties encountered: Issues related to school meals, outings, vacations, monotony, snacking, satiety, reconstitution constraints, and digestive symptoms.

Impact and follow‐up: Symptom improvement, use of NGT at school, enrollment in a therapeutic program, and dietary and psychological support.

Long‐term consequences: Development of feeding difficulties: selectivity, texture issues, nausea, behavioral disorders, altered taste, and social difficulties.

Personal opinion: Recommendation and willingness to undergo the treatment again.

### 2.2 Statistics

Proportions are given as absolute values accompanied by percentages. Continuous variables are expressed as median, interquartile range, and minimum–maximum range.

## 3 RESULTS

### 3.1 Characteristics of patients at inclusion

**Figure 1:**
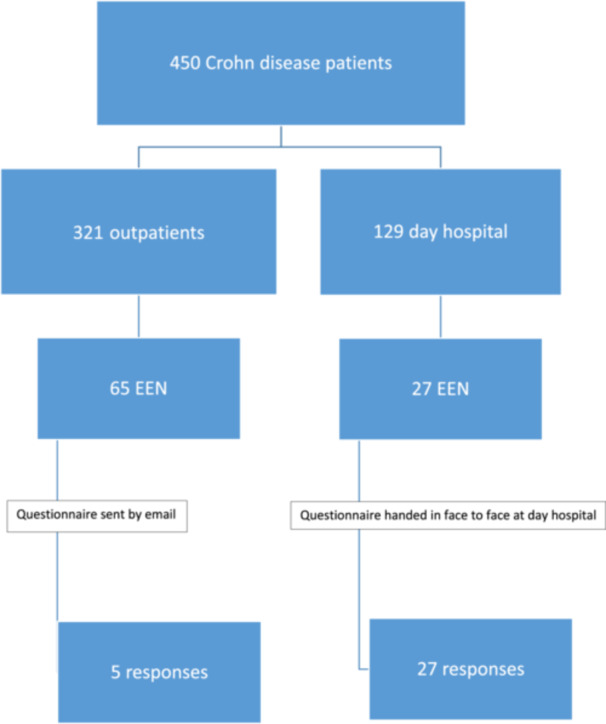
Flow chart.

The characteristics of the patients' recruitment are detailed in Figure 1 and Table 1.

**Table 1:** Patient demographics at diagnosis.

Demographics at diagnosis	*n* = 32
Female, *n* (%)	10 (32)
Paris classification	
Age at diagnosis	
A1a	5 (16)
A1b	27 (84)
Location	
L1	8 (25)
L2	8 (25)
L3	16 (50)
L4a	17 (53)
L4b	1 (3)
Behavior	
B1	24 (75)
B2	8 (25)
B3	0
P	1 (3)
Growth	
G0	27 (84)
G1	5 (16)
Extra‐intestinal manifestations	5 (16)

Twenty percent (*n* = 92) of the patients followed up for CD received EEN. Of these, 32 responded to the questionnaire, resulting in a response rate of 35%. All patients were followed up for CD, with 22 (69%) of them being boys. The median age at the start of EEN was 12.5 years (range: 12.5–13.25; min–max: 6–16 years). This was the first treatment for CD in 24 patients (75%), which was initiated during hospitalization in 87% of the cases (*n* = 28). The median treatment duration was 7 weeks (range: 4–12; min–max: 1–24 weeks). Initially, 23 patients (72%) received oral treatment, while 9 (28%) required NGT from the outset. Three patients were unable to continue oral intake, which necessitated NGT placement. Ultimately, 20 patients (62%) completed the entire nutritional treatment orally, while 12 (38%) required NGT. The median time between treatment and response to the questionnaire was 5 years (range: 3–7.25; min–max: 0–12 years).

### 3.2 Results during treatment

Forty‐one percent (*n* = 13) of the patients took their liquid diet together with their families, and at the time, family members were having their regular meals.

All patients (100%, *n* = 32) encountered eating difficulties during EEN, and 23 experienced multiple issues. The main difficulties were related to eating: desire to eat (*n* = 16), liquid‐only diet (*n* = 14), monotony (*n* = 14), and taste of milk (*n* = 4). Social difficulties included outings with friends (*n* = 7), school cafeterias (*n* = 7), and vacations (*n* = 7). Practical issues such as product reconstitution (*n* = 8), adherence to dosages (*n* = 2), or discomfort related to the NGT (*n* = 3) were noted. Additionally, digestive symptoms, such as diarrhea and bloating, persisted (*n* = 8).

Among the 32 patients, 38% (*n* = 12) discontinued the treatment. The primary cause of discontinuation was treatment intolerance in three quarters of the cases and medical reasons in one quarter. Intolerance was due to aversion (*n* = 2), inability to tolerate NGT (*n* = 2), vomiting (*n* = 2), and diarrhea (*n* = 1). Two patients stopped for social reasons: the school cafeteria meals were too stigmatizing, and maintaining a social life with friends was challenging. Among these 12 patients, one third (*n* = 4) had received dietary follow‐up, 2 received psychological support, and 8 of the 12 did not eat meals with their families.

The average time for patients to notice symptom improvement was 6 weeks (range: 2–12 weeks).

During their treatment, 20 patients (62%) received support from the multidisciplinary team: 3 participated in the therapeutic education program, 13 received nutritional follow‐up, and 5 received psychological support.

### 3.3 Results post‐EEN

Ten patients (32%) reported persistent eating difficulties a median of 5 years after nutritional treatment. One third (*n* = 4) of the participants met the three criteria for difficulty. Among these 10 patients, 7 had adhered to the treatment without premature discontinuation, and 4 did not eat meals with their families. Three patients reported taste changes that made certain foods unpalatable. Three patients needed to split meals, with one feeling that their stomach had shrunk. Two patients developed ED: one with hyperphagia and one with selective eating. Two patients had issues with milk, one had difficulties with food textures, and the other had chewing problems. In addition to these eating difficulties, four patients experienced long‐term social difficulties related to isolation during meals. Among the 13 patients who received dietary follow‐up during treatment, 6 developed eating or social difficulties related to food posttreatment. None of the five children who received psychological support during nutritional treatment developed long‐term issues.

### 3.4 Evaluation of recommendations and re‐treatment with EEN

Fifty‐nine percent of the patients (*n* = 19) recommended EEN. The main reason for this was that it avoided other treatments and their side effects. Additionally, patients appreciated its rapid efficacy and ability to alleviate the disease. It also helped to avoid medication use and manage the disease. The natural essence of EEN is another advantage. Furthermore, EEN helped in weight gain, increased energy, improved symptoms, and reduced pain.

**Figure 2:**
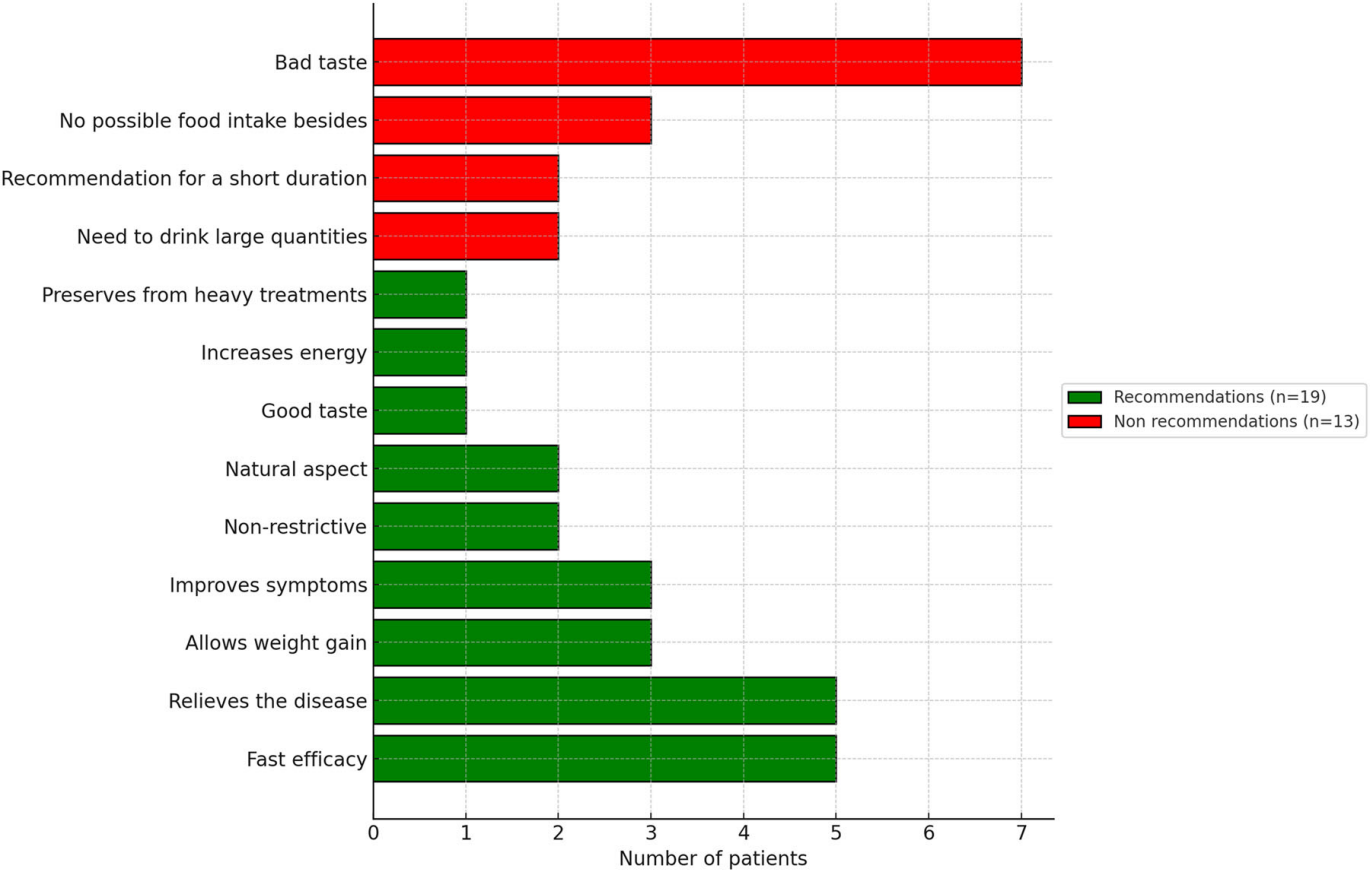
Reasons to recommend or not EEN. EEN, exclusive enteral nutrition.

Forty‐one percent (*n* = 13) would not recommend it, with the main reason for 46% (*n* = 5) being its bad taste, often described as “like spoiled milk” and nauseating. It was necessary to drink large quantities (often leading to the use of an NGT), and no other food was available, which was mentally challenging. Some specified that they might recommend it for short‐term treatment with dosage adjustment and for young children who have not yet developed their palate. The reasons for this are illustrated in Figure 2.

Forty‐one percent (*n* = 13) of the patients would accept undergoing EEN again for several reasons:

For five of them, out of resilience and as a last resort, accepting the treatment out of necessity, with strong mental willpower, a sense of obligation, and if no other option was effective. Patients frequently used expressions like “if I have no choice” or “it's part of me.”

1. For three of them, for the nutritional effects: weight and muscle gain, positive nutritional benefits (one patient consumed EEN after weightlifting sessions for the protein intake).
2. Control of disease without medication.

**Figure 3:**
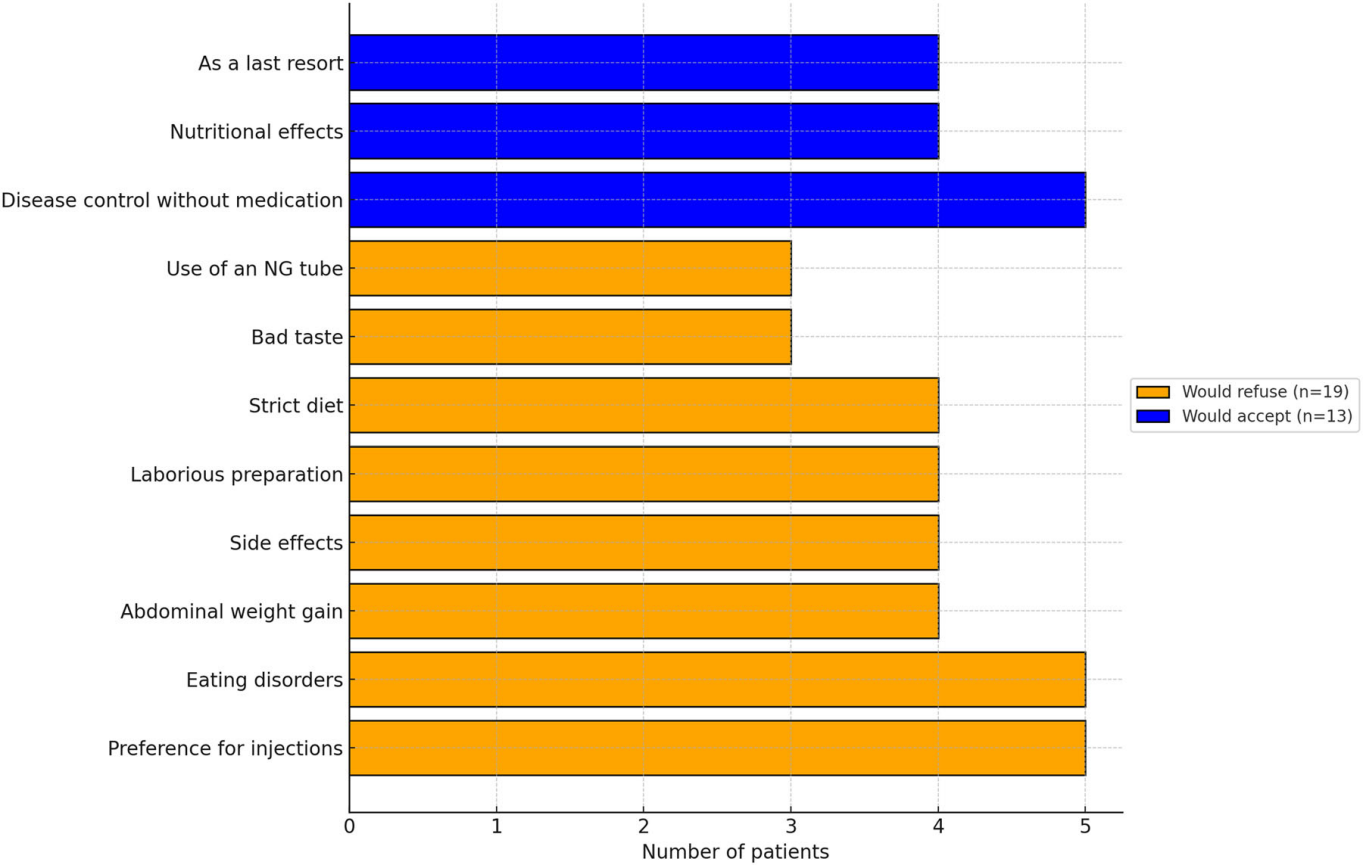
Reasons to accept or not another EEN course. EEN, exclusive enteral nutrition; NG, nasogastric.

Fifty‐nine percent (*n* = 19) who would not undergo EEN again cited reasons such as NGT, taste, inability to eat anything else, and the development of ED posttreatment. Twenty‐two percent (*n* = 4) preferred biotherapy, as it did not affect their quality of life. The reasons for this are illustrated in Figure 3.

## 4 DISCUSSION

Among all patients actively followed up for CD in the department, 20% received EEN. All patients experienced difficulties related to diet or social life during this treatment despite multidisciplinary team support in 62% of the cases. Additionally, 39% prematurely discontinued treatment, while 32% continued to experience eating difficulties after treatment, such as taste alterations, chewing problems, food texture issues, or ED. Nevertheless, 59% would recommend EEN for medical or nutritional reasons, yet paradoxically, 59% would not agree to undergo treatment a second time. Tolerance to EEN is critical, especially in children. Indeed, 100% (*n* = 32) of the patients experienced dietary difficulties during treatment, which impacted their diet or social life. Ashton et al.^12^ emphasized the importance of tolerance to the effectiveness of EEN in CD. Moreover, the study results indicate that the poor taste of EEN is a major barrier to adherence, which is consistent with observations by Brown et al.,^4^ who identified palatability as a crucial factor. Isolation from family meals or the feeling of exclusion from peer life is another significant limitation of treatment, as noted by Brown and Svolos.^4^, ^11^ Future research could focus on developing alternative formulations to mitigate the negative side effects of EEN, as suggested by Hudson,^12^ one of which is the Crohn's Disease Exclusion Diet, which combines EEN with solid food or CD‐treat diet.^13^, ^14^ This approach addresses the desire expressed by the parents of children with CD.^5^, ^9^, ^11^ Additionally, it would be beneficial to propose taste improvements for the product to reduce aversion in line with Godala's recommendations.^15^ Despite these challenges, 41% of the patients took their liquid diet together with their families; at the time, family members were having their regular meals. This shows that, even with a monotonous diet, a significant portion of patients value the time spent with their loved ones during meals, highlighting the importance of the social and emotional aspects of meals beyond mere nutrition. The ability to maintain normal social interactions can play a crucial role in the overall well‐being of patients and their adherence to treatment. It is also essential to consider each patient's personality traits and the importance of their surroundings, as specified by Walls.^3^ Premature discontinuation of treatment in 39% of patients confirms the adherence issues already observed by Gkikas and Jatkowska.^8^, ^16^ Interestingly, among the 12 patients who prematurely discontinued nutritional treatment, 8 did not have family meals, underscoring the importance of family mealtime. The study data revealed significant disparities in access to various types of support offered by multidisciplinary teams. 62% of patients received multidisciplinary team support during treatment. Only three patients participated in the therapeutic education programs, which may indicate a lack of information or access to these programs or a possible underestimation of their importance by patients. Furthermore, 13 patients received support from a dietitian‐nutritionist, indicating that nutritional support is relatively well integrated into the care pathway, although improvements are still needed to ensure equitable access for all patients. Multidisciplinary and dietary teams play a crucial supporting role in this treatment.^4^, ^5^, ^10^, ^12^, ^17^ Only five patients (15%) received psychological support during treatment. Eiser and Morse^18^ noted that psychological support is crucial for children with chronic diseases as it helps them manage the mental and emotional aspects of treatment, with anxiety and depression tending to improve along with disease remission.^19^ It is interesting to note that among our patients, four (12.5%) experienced social difficulties, such as isolation, induced by nutritional treatment. Examining long‐term tolerance, the study revealed that 32% or one third of the patients reported persistent eating difficulties after stopping treatment, including ED, such as binge eating or food selectivity, food texture issues, chewing problems, and taste alteration. The majority of these patients (7 of the 10 with difficulties) completed nutritional treatment. The average time between the questionnaire and EEN was 5.6 years. This aspect has never been previously described, as studies have primarily focused on immediate tolerance. Gavin et al.^9^ recommend longitudinal studies to evaluate the prolonged effects of nutritional treatment. In this context, where nutrition plays a crucial role in the perception of the disease and its relapses, eliminating all solid food for 8 weeks could indeed lead to ED in these patients. Patients with IBD consider diet as an element that influences the course of the disease. About 16%–51% even believe that certain foods can trigger the disease or cause a relapse,^15^, ^20^ which can lead to an exclusion diet potentially causing deficiencies. In this context, it might be pertinent to establish a psychological profile of patients suitable for receiving nutritional treatment without developing ED in the long term and to implement appropriate psychological support, even a joint psychologist–dietitian consultation before treatment, to assess the risk factors for subsequent ED and propose an alternative treatment in this context.^3^ In our study, among the 13 patients who received nutrition, 6 developed distant eating or social difficulties related to food, while among the 5 patients who received psychological follow‐up, none developed a distant disorder, highlighting the role of this type of support.^4^, ^5^, ^10^, ^12^, ^17^, ^18^ Fifty‐nine percent of patients would recommend the treatment because of its rapid effectiveness, nutritional benefits, and avoidance of the need for treatments and their side effects, in line with published data^4^, ^11^: the expected benefits outweigh the constraints. Forty‐one percent of patients indicated that they would be willing to undergo EEN again, especially for its potential to control the disease without medication and its positive nutritional effects, such as weight and muscle gain. This duality in recommendations is consistent with Connors' observations,^7^ who noted that EEN avoids the long‐term use of corticosteroids, but presents challenges in terms of treatment adherence. Four study patients preferred biological therapies to nutritional treatment, noting that it did not affect their quality of life. With anti‐tumor necrosis factor, patients say that they can eat whatever they want and rediscover the pleasure of eating.^21^

It is crucial to note that this study focused on a pediatric population, patients who often have no choice but to follow the directives of doctors and parents. Despite these constraints, these children showed remarkable resilience. They must cope with the strict demands of the treatment, including adhering to a liquid diet and using an NGT, while continuing their daily activities and maintaining a certain quality of life. Resilience is a fundamental aspect that deserves to be highlighted and considered in the overall evaluation of EEN tolerance and acceptance. The main strengths of our study are as follows: the use of a detailed questionnaire and direct follow‐up with patients and their families ensured rich and precise data collection. Although limited in size, the sample included various experiences representing a range of perspectives. The main weaknesses of this study are sample size and selection bias. With only 32 responses out of the 92 actively followed‐up patients who received EEN, the sample was limited, potentially affecting the generalization of the results. Patients responding to the questionnaire may have had specific experiences, introducing a selection bias. Moreover, a few outpatients returned the questionnaire. Face‐to‐face interviews allow for more data collection than impersonal questionnaires. Moreover, the average time between treatment and response to the questionnaire was 5.6 years (range: 0–12 years). This is a broad range, and it could have affected the responses obtained to some of the questionnaire items, that is, it is more likely for a child filling the questionnaire straight after a course of EEN to have vivid memories of the difficulties they had to go through, while they may not have seen the benefits yet, compared to a child filling the questionnaire 12 years later.

## 5 CONCLUSION

Despite the challenges and constraints associated with EEN treatment, a significant proportion of pediatric patients recognize its benefits and effectiveness in achieving early remission. However, this treatment can lead to ED and requires particular attention. It is essential to provide support tailored to the individual needs of patients, including therapeutic alternatives, as well as dietary and psychological support, to improve treatment adherence and prevent the development of ED. Personalized interventions based on the specific needs of patients can enhance adherence to and outcomes of EEN.

## CONFLICT OF INTEREST STATEMENT

The authors declare no conflicts of interest.

## Data Availability

Data supporting this study are included in the article.
